# Analysis of Methanolic Extracts and Crude Polysaccharides from the Leaves of *Chuanminshen violaceum* and Their Antioxidant Activities

**DOI:** 10.3390/antiox8080266

**Published:** 2019-08-01

**Authors:** Shang Lin, Hong-Yi Li, Zi-Ying Wang, Xin Liu, Yang Yang, Zheng-Wen Cao, Gang Du, Li Zhao, Qing Zhang, Ding-Tao Wu, Wen Qin

**Affiliations:** 1 Institute of Food Processing and Safety, College of Food Science, Sichuan Agricultural University, Ya’an 625014, China; 2 Sichuan Provincial Institute for Food and Drug Control, Chengdu 611730, China

**Keywords:** *Chuanminshen violaceum*, phenolic compound, polysaccharide, chemical structure, antioxidant activity

## Abstract

The root of *Chuanminshen violaceum* is used as an important edible and medicinal plant in China. However, its leaves are generally considered byproducts, and therefore do not have a use. Thus, the phenolic compounds in the methanolic extracts (CVLMs) and the chemical characteristics of crude polysaccharides (CVLPs) from the leaves of *C. violaceum* and their in vitro antioxidant activities were explored. The results showed that chlorogenic acid and rutin were the major individual phenolic compounds in the leaves, which ranged from 1.22 ± 0.03 to 2.87 ± 0.04 mg/g DW, and from 2.25 ± 0.04 to 4.03 ± 0.05 mg/g DW, respectively. Meanwhile, the extraction yields of CVLPs from the leaves ranged from 4.73% to 5.41%. The CVLPs consisted of mannose, rhamnose, galacturonic acid, glucose, galactose, and arabinose, suggesting the existence of pectic polysaccharides. Furthermore, both CVLMs and CVLPs exhibited strong antioxidant activities. Chlorogenic acid and rutin were major contributors to the antioxidant activities of CVLMs, and the antioxidant activities of CVLPs were closely correlated to their α-1,4-D-galactosiduronic linkages. The results are beneficial for understanding the chemical properties and in vitro antioxidant activities of CVLMs and CVLPs. The leaves of *C. violaceum* have potential to be developed as natural antioxidants.

## 1. Introduction

Generally, oxidative stress in the human body is related to various diseases/disorders, such as metabolic, neurodegenerative, cardiovascular, mitochondrial diseases, and even cancer [1]. Recently, it has been reported that antioxidants are effective in controlling the production of free radicals, and are also helpful in detoxifying activities [1,2]. Various synthetic antioxidants, such as butylated hydroxyanisole (BHA), butylated hydroxytoluene (BHT), and tert-butyl hydroquinone (TBHQ) have been commonly used to reduce reactive oxygen species (ROS) damage in many fields, such as the food industry and biomedicines [3,4,5]. However, their toxic and carcinogenic side effects, shown in animal models, are affecting their acceptability from consumers [6]. Thus, due to their relatively lower toxicity and side effects, there is an increasing demand for natural antioxidants from plants, such as phenolic compounds and polysaccharides [2,7,8,9].

*Chuanminshen violaceum* is a species of a monotypic genus in the family Umbelliferae [10], which is mainly an economic plant in four heavily cultivated regions, including Chengdu, Langzhong, Bazhong, and Guangyuan City, Sichuan Province, China. The root of *C. violaceum* has been used as an edible and medicinal plant for a long time by the local people [11]. Additionally, due to the high amounts of phenolic compounds and polysaccharides, and the strong antioxidant activities [8,11], *C. violaceum* is considered a natural source of antioxidants. However, the applications of *C. violaceum* are limited to its roots, and the leaves are usually regarded as byproducts. The leaves of *C. violaceum* are generally used as forage for livestock by local people, or may even be simply abandoned. In fact, about a half of a *C. violaceum* plant is composed of leaves, which suggests a high development potential. Furthermore, to the best of our knowledge, the bioactive compounds, such as phenolic compounds and polysaccharides, from the leaves of *C. violaceum* have seldom been studied. Phenolic compounds and polysaccharides from natural sources exert strong antioxidant activities [2,7,8]. Therefore, the study of phenolic compounds and polysaccharides, and their related in vitro antioxidant activities, from the leaves of *C. violaceum* is meaningful and important. Meanwhile, it is also helpful to better understand their chemical characteristics, and to increase the possibility of their use as natural antioxidants in industrial applications.

In the present work, the phenolic compounds in the methanolic extracts and the chemical characteristics of crude polysaccharides from the leaves of *C. violaceum* and their in vitro antioxidant activities were explored. Furthermore, in order to better understand the structure–bioactivity relationship of CVLPs, the correlations between their in vitro antioxidant activities and their glycosidic linkages were also studied.

## 2. Materials and Methods

### 2.1. Materials and Chemicals

Four batches of leaves of *C. violaceum* were collected from Chengdu, Langzhong, Bazhong, and Guangyuan City, Sichuan Province, China. Then, samples were dried and milled into powders. Subsequently, the powders were passed through a 60-mesh size screen and stored at −20 °C for further analysis.

Gallic acid, rutin, 2,2-diphenyl-1-(2,4,6-trinitrophenyl) hydrazyl (DPPH), chlorogenic acid, Folin–Ciocalteu reagent, 2,4,6-tri(2pyridyl)-s-triazine (TPTZ), 2,2′-azino-Bis (3-ethylbenzthiazoline-6-sulphonic acid) (ABTS), 1-phenyl-3-methyl-5-pyrazolone (PMP), sodium nitroprusside (SNP), phosphoric acid, sulfanilamide, 6-hydroxy-2,5,7,8-tetramethyl chroman-2-carboxylic acid (Trolox), and monosaccharide standards (rhamnose, mannose, galacturonic acid, glucuronic acid, galactose, glucose, xylose, and arabinose) were purchased from Sigma-Aldrich (St. Louis, MO, USA).

### 2.2. Preparation of Methanolic Extracts and Crude Polysaccharides from the Leaves of C. violaceum

The methanolic extracts and crude polysaccharides from the leaves of *C. violaceum* were extracted according to our previous methods with some modifications [2,7]. In brief, 5.0 g of sample powders were extracted with 150 mL of 80% methanol (*v*/*v*) by an ultrasound processer (480 W, 24 KHZ, Kangshijie Ultrasonic Wave Tech., Dongguan, China) at 60 °C for 80 min. Then, centrifugation at 4000× *g* was performed for 20 min. The residues were collected for further extraction of crude polysaccharides, and the supernatant was subsequently concentrated by a rotary evaporator at 45 °C (RE-52AA, Yarong Company, Shanghai, China). Afterwards, the methanolic extracts from the leaves of *C. violaceum* (CVLMs) collected from Chengdu, Langzhong, Bazhong and Guangyuan were marked as CVLM-A, CVLM-B, CVLM-C and CVLM-D, respectively. Samples were stored at −20 °C for further HPLC analysis and antioxidant activity assay.

Then, the collected residues were used to extract the crude polysaccharides in *C. violaceum* (CVLPs). Briefly, the residues were extracted twice by 150 mL water at 95 °C for 2 h. After centrifugation (4500× *g* for 20 min), the supernatant was combined and collected. The combined extracts were concentrated under a vacuum at 60 °C. Then, 3.0 mg of heat-stable α-amylase (40,000 U/g, peak enzymatic activity at 80 °C) was added into mixtures to remove the starch at 80 °C for 2 h. Subsequently, the temperature was increased to 100 °C to inactivate the enzymes. Three volumes of 95% (*v*/*v*) ethanol were used for the precipitation of the crude polysaccharides in the supernatant at 4 °C overnight. After centrifugation (4500 × g for 20 min), the precipitations were dissolved in water and freeze dried. Afterwards, the CVLPs extracted from the leaves of *C. violaceum* collected from Chengdu, Langzhong, Bazhong, and Guangyuan were marked as CVLP-A, CVLP-B, CVLP-C and CVLP-D, respectively, and then stored at −20 °C for further analysis.

### 2.3. Chemical Analysis of Methanolic Extracts from the Leaves of C. violaceum (CVLMs)

#### 2.3.1. Determination of Total Phenolic Content and Total Flavonoid Content

Total phenolic content (TPC) and total flavonoid content (TFC) of CVLM-A, CVLM-B, CVLM-C and CVLM-D were determined by calorimetry according to our previous reported methods [12]. Afterwards, the TPC and TFC of CVLMs were expressed as mg GAE/g DW and mg RE/g DW, respectively.

#### 2.3.2. HPLC Analysis of Individual Phenolic Compounds

The HPLC analysis of individual phenolic compounds of CVLM-A, CVLM-B, CVLM-C and CVLM-D were performed by our previous reported method [2]. An Agilent 1260 series HPLC (Agilent Technologies, Palo Alto, CA, USA) equipped with a diode-array detector (DAD, Agilent Technologies, Palo Alto, CA, USA) and a ZORBAX Eclipase XDB-C18 column (250 mm × 4.6 mm, 5 µm) was utilized for the analysis of each sample. The chromatographic separation was performed by a gradient elution with 0.5% (*v*/*v*) acetic acid solution (A) and acetonitrile (B) at 25 °C at a flow rate of 0.8 mL/min. Then, 20 µL of each sample was eluted as follows: 0 min, 5% B; 5 min, 5% B; 50 min, 5–20% B; 60 min, 20–70%; 65 min, 70–5% B. Identification of the major individual phenolic compounds of CVLMs was carried out by comparing retention times and absorption spectra of commercial standards. The identified compounds were quantified by using calibration curves of the standards (chlorogenic acid and rutin). Afterwards, the content of each individual phenolic compound was expressed as milligram per gram dry weight of a *C. violaceum* plant (mg/g DW).

### 2.4. Characterization of Crude Polysaccharides from the Leaves of C. violaceum (CVLPs)

#### 2.4.1. Chemical Composition Analysis

The contents of total polysaccharides in CVLP-A, CVLP-B, CVLP-C, and CVLP-D were determined by phenol-sulfuric methods, while the glucose was used as a standard [13]. The contents of total uronic acids in CVLPs were determined by the m-hydroxydiphenyl method, while the galacturonic acid (GalA) was used as a standard [14]. Furthermore, the contents of total proteins in CVLPs were determined by Bradford’s method, while the bovine serum albumin was used as a standard [15].

#### 2.4.2. Determination of Molecular Weights

The molecular weights (*M_w_*) and polydispersities (*M_w_*/*M_n_*) of CVLP-A, CVLP-B, CVLP-C and CVLP-D were measured by high performance size exclusion chromatography coupled with multi angle laser light scattering and a refractive index detector (HPSEC-MALLS-RID, Wyatt Technology Co., Santa Barbara, CA, USA) according to our previously reported method with minor modifications [16]. The TSK-Gel GMPWXL (300 mm × 7.8 mm) was utilized for the separation of CVLPs at 30 °C. The *dn/dc* value of CVLPs was selected as 0.150 mL/g according to a previous study [17]. Meanwhile, the Astra software (version 7.1.3, Wyatt Technology Co., CA, USA) was utilized for data acquisition and analysis.

#### 2.4.3. Determination of Constituent Monosaccharides

The constituent monosaccharides of CVLP-A, CVLP-B, CVLP-C and CVLP-D were analyzed by HPLC according to our previous methods with minor modifications [7]. In brief, 4.0 mg of each sample was hydrolyzed with 2.0 M trifluoracetic acid (TFA) at 95 °C for 6 h. Subsequently, the hydrolyzates were used for PMP derivatization. Meanwhile, a standard solution, including rhamnose, mannose, glucuronic acid, glucose, galacturonic acid, galactose, xylose, and arabinose, was also derivatized by PMP. Finally, an Agilent 1260 series LC system (Agilent Technologies, Palo Alto, CA, USA) coupled with a ZORBAX Eclipse XDB-C18 column (4.6 × 250 mm i.d. 5 µm) was utilized for the analysis of PMP derivatives. The mobile phase was a mixture of phosphate buffer solution (0.1 M, pH = 6.7) and acetonitrile (83:17, *v*/*v*). The flow rate and the wavelength of DAD were set at 1.0 mL/min and 245 nm, respectively.

#### 2.4.4. Fourier Transform Infrared (FT-IR) Analysis

The FT-IR analysis of CVLP-A, CVLP-B, CVLP-C and CVLP-D was also performed by our previously reported method [7]. The Nicolet iS 10 FT-IR Spectrometer (Thermo Fisher scientific, Waltham, MA, USA) was used to record the IR spectra of CVLPs in the frequency range of 4000–500 cm^−1^.

### 2.5. Evaluation of In Vitro Antioxidant Activities of CVLMs

The DPPH radical scavenging activities, ABTS radical scavenging activities, and ferric-reducing antioxidant powers (FRAPs) of CVLM-A, CVLM-B, CVLM-C and CVLM-D were determined according to our previously reported methods [2,12]. Methanol was used as the blank control, and Trolox was used as the positive standard. Afterwards, the DPPH radical scavenging activities, ABTS radical scavenging activities, and FRAPs of each sample were expressed as µmol Trolox equivalent per gram of dry weight of *C. violaceum* plant (µmol Trolox/g DW).

### 2.6. Evaluation of In Vitro Antioxidant Activities of CVLPs

#### 2.6.1. Determination of In Vitro Antioxidant Activities

The ABTS radical scavenging activities and nitric oxide (NO) radical scavenging activities of CVLP-A, CVLP-B, CVLP-C and CVLP-D were also determined according to our previous method [7]. Butylated hydroxytoluene (BHT) was used as the positive control. The ABTS and NO radical scavenging activities of CVLPs were measured at five different concentrations each, and the IC_50_ values (mg/mL) of CVLPs were calculated based on a logarithmic regression curve [7].

Furthermore, the FRAPs of CVLP-A, CVLP-B, CVLP-C and CVLP-D were determined according to our previous method [7]. Butylated hydroxytoluene (BHT) was used as the positive control, and the FRAPs of CVLPs were expressed as the absorbance at 593 nm.

#### 2.6.2. Effects of Partial Acid Hydrolysis and Enzymatic Degradation on the In Vitro Antioxidant Activites of CVLPs

Partial acid hydrolysis of CVLP-A was performed by a previous method with minor modifications [18]. Briefly, 20.0 mg of each sample was hydrolyzed by 1.0 M trifluoroacetic acid at 90 °C for 4 h. After hydrolysis, the partial acid hydrolysates of CVLPs were evaporated to dryness at 60 °C under vacuum, and washed by methanol to remove the TFA. The dried hydrolysates were dissolved in pure water, and subsequently freeze dried. The dried sample was marked as CVLP-A2 and stored at −20 °C for further analysis.

Furthermore, enzymatic degradation of CVLP-A was also performed by a previous method with minor modifications [18]. Briefly, 4.0 mL of each sample (5 mg/mL) was mixed with 10 mg of pectinase (1 U/mg), and incubated at 40 °C for 10 h. Subsequently, the pectinase was inactivated at 90 °C for 1 h, and the mixture was centrifuged at 4000× *g* for 20 min. The supernatant was freeze dried, and the dried sample was marked as CVLP-A3 and stored at −20 °C for further analysis.

Afterwards, the in vitro antioxidant activities of CVLP-A2 and CVLP-A3 were determined according to the methods of Section 2.6.1.

### 2.7. Statistical Analysis

All experiments were conducted in triplicate, and data were expressed as means ± standard deviations. Origin 9.0 software (OriginLab Corporation, Northampton, Mass., USA) and S.P.S.S. 11.0 software (IBM Analytics, IBM, Armonk, NY, USA) were used for statistical analysis. Statistical significances were carried out by one-way analysis of variance (A.N.O.V.A.) and Duncan’s test. Values of *p* < 0.05 were considered as statistically significant.

## 3. Results and Discussions

### 3.1. Major Chemical Compositions of CVLMs

The total phenolic content (TPC), total flavonoid content (TFC) and the contents of major individual phenolic compounds identified in the leaves of *C. violaceum* are summarized in Table 1. Results showed that the TPC and TFC in the leaves of *C. violaceum* collected from different regions in China ranged from 8.02 ± 0.27 to 12.55 ± 1.01 mg GAE/g DW, and from 5.36 ± 0.22 to 7.97 ± 0.31 mg RE/g DW, respectively. Meanwhile, the TPC in the leaves of *C. violaceum* were higher than that of *C. violaceum* roots (7.88 mg GAE/g DW), and other commercial tea sources such as bitter gourd, Qingke, *Camellia japonica* L., *Lilium brownie*, *Siraitia grosvenorii*, *Agadtacge rygisa*, and *Amomum villosum* Lour [12,19,20,21]. The results suggest that the leaves of *C. violaceum* are rich sources of natural phenolic compounds. Furthermore, in order to better understand the major individual phenolic compounds in methanolic extractions from the leaves of *C. violaceum*, the HPLC-DAD analysis was performed according to a previously reported method [2]. Chlorogenic acid and rutin were identified as two major individual phenolic compounds in CVLMs according to the HPLC analysis. Meanwhile, the contents of chlorogenic acid and rutin in CVLM-A, CVLM-B, CVLM-C and CVLM-D ranged from 1.22 ± 0.03 to 2.87 ± 0.04 mg/g DW, and from 2.25 ± 0.04 to 4.03 ± 0.05 mg/g DW, respectively. The levels of chlorogenic acid and rutin are relatively higher than those of other rich sources of natural phenolic compounds, such as *Camellia japonica*, *Bombaxceiba*, *Chaenomeles sinensis*, *Eriobotrya japonica*, and *Chrysanthemum morifolium* [20]. Moreover, it has been reported that chlorogenic acid and rutin are related to various bioactivities, such as antioxidant, anti-cancer, and anti-diabetic activities [22,23,24].

### 3.2. Chemical Characterizations of CVLPs

#### 3.2.1. Chemical Compositions of CVLPs

The extraction yields and chemical compositions of CVLP-A, CVLP-B, CVLP-C and CVLP-D are shown in Table 2. The extraction yields of CVLPs from the leaves of *C. violaceum* collected from different regions in China ranged from 4.73% to 5.41%. In addition, the contents of total polysaccharides in CVLPs ranged from 80.92% to 82.08%, while few proteins were detected in CVLPs (range from 1.99% to 2.73%). The results suggested that polysaccharides were major components of CVLPs. Furthermore, the levels of total uronic acids of CVLPs ranged from 22.03% to 30.96%. The relatively high amount of uronic acids in CVLPs suggested the existence of pectic-like polysaccharides in the leaves of *C. violaceum* [7,16,25]. Indeed, uronic acids in natural polysaccharides are believed to correlate with their various bioactivities, such as their antioxidant activity [7,25].

#### 3.2.2. Molecular Weights and Constituent Monosaccharides of CVLPs

**Figure 1:**
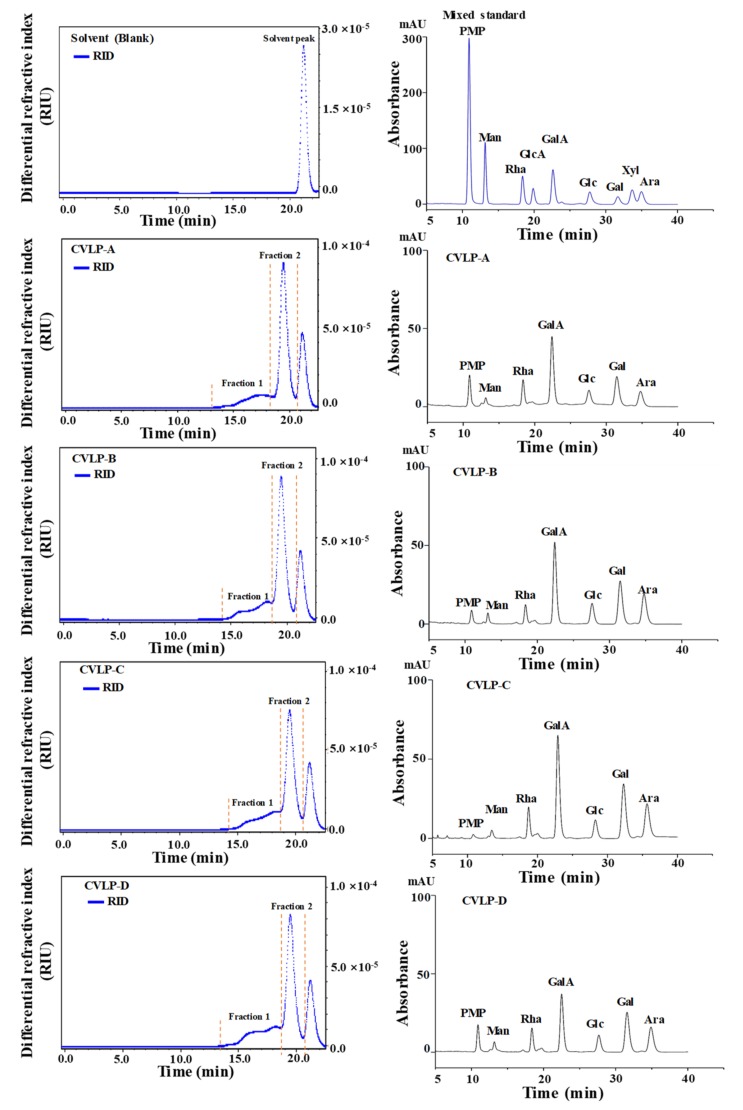
High performance size exclusion chromatograms (**left**) and high-performance liquid chromatography profiles (**right**) of CVLPs. **CVLP-A**, **CVLP-B**, **CVLP-C and CVLP-D**, crude polysaccharides extracted from the leaves of *C. violaceum* collected from Chengdu, Langzhong, Bazhong and Guangyuan, respectively; **PMP**, 1-phenyl-3-methyl-5-pyrazolone; **Man**, mannose; **Rha**, rhamnose; **GalA**, galacturonic acid; **Glc**, glucose; **Gal**, galactose; **Ara**, arabinose.

Generally, antioxidant activities of natural polysaccharides are correlated with their structural features, such as molecular weights and constituent monosaccharides [7,26]. Thus, it is necessary to analyze the structures of CVLPs, which is helpful for understanding their structure–bioactivity relationships. Therefore, the molecular weights and constituent monosaccharides of CVLPs were determined, and the results are summarized in Figure 1. As shown in Figure 1, two fractions of polysaccharides (fraction 1 and fraction 2) were detected in the CVLPs, while the fraction from 21 to 22 min was the solvent peak. Results indicated that CVLPs were heteropolysaccharides. Furthermore, Table 2 shows the detailed molecular weights of polysaccharide fraction 1 and 2 of CVLPs from the leaves of *C. violaceum* collected from different regions, which ranged from 5.37 × 10^4^ to 9.45 × 10^4^ Da, and from 1.01 × 10^4^ to 1.21 × 10^4^ Da, respectively. In addition, the polydispersities of polysaccharide fraction 1 and 2 ranged from 1.43 to 2.00, and from 1.04 to 1.31, respectively. Fraction 1 of CVLPs showed a relatively wider molecular weight distribution than that of fraction 2, which was in accordance with HPSEC chromatograms.

The constituent monosaccharides of CVLPs from the leaves of *C. violaceum* collected from different regions were analyzed by the HPLC system. The HPLC-DAD profiles of CVLP-A, CVLP-B, CVLP-C, and CVLP-D are shown in Figure 1. Results indicated that the constituent monosaccharides of CVLPs consisted of mannose, rhamnose, galacturonic acid, glucose, galactose, and arabinose, which further confirmed that there are pectic-like polysaccharides in the leaves of *C. violaceum*. The molar ratios of constituent monosaccharides in CVLPs are shown in Table 3.

#### 3.2.3. FT-IR spectra of CVLPs

**Figure 2:**
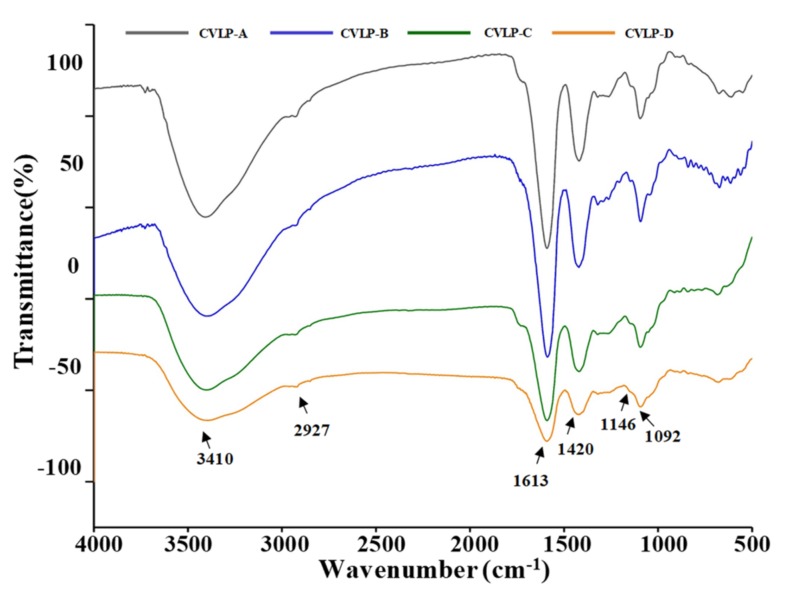
Fourier transform infrared (FT-IR) spectra of CVLPs. **CVLP-A**, **CVLP-B**, **CVLP-C and CVLP-D**, crude polysaccharides extracted from the leaves of *C. violaceum* collected from Chengdu, Langzhong, Bazhong and Guangyuan, respectively.

In order to investigate the chemical structures of CVLPs, the FT-IR spectrum between 4000 and 500 cm^−1^ was performed, and the results are shown in Figure 2. The intense and broad bands at 3410 cm^−1^ are characteristic of hydroxyl groups [27]. The peak at 2927 cm^−1^ was assigned to C–H asymmetric stretching vibration [28]. In addition, the peak at 1613 cm^−1^ was the C=O asymmetric stretching of –COO, which further confirmed that uronic acids existed in CVLPs [29,30]. The band at 1420 cm^−1^ was attributed to the bending vibration of C–H or O–H [31,32]. In addition, the band at 1146 cm^−1^ was the asymmetric C–O–C stretching vibration, suggesting the presence of –OCH_3_ [33]. No signal was detected at 1555 cm^−1^, which confirmed a very low amount of proteins in CVLPs [34].

### 3.3. In Vitro Antioxidant Activities of CVLMs

#### 3.3.1. In Vitro Antioxidant Activities

**Figure 3:**
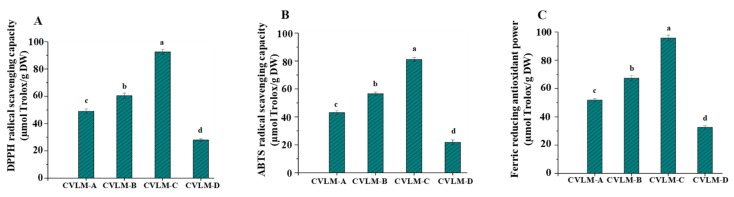
DPPH radical scavenging activities (A), ABTS radical scavenging activities (B) and ferric reducing antioxidant powers (C) of CVLMs. **CVLM-A**, **CVLM-B**, **CVLM-C** and **CVLM-D**, methanolic extractions from the leaves of *C. violaceum* collected from Chengdu, Langzhong, Bazhong and Guangyuan, respectively. Values represent mean ± standard deviation, and superscripts a–d differ significantly (*p* < 0.05) among CVLMs collected from different regions. Statistical significances were determined by A.N.O.V.A.

Generally, plants rich in phenolic compounds exert strong antioxidant activities [2,35,36,37]. In addition, many studies have shown that the leaves of various plants usually exhibit remarkable antioxidant activities due to their high amounts of phenolic compounds [38,39,40]. Thus, it is important to evaluate the in vitro antioxidant activities of leaves of *C. violaceum*. Therefore, the in vitro antioxidant activities, including DPPH radical scavenging activities, ABTS radical scavenging and FRAPs of CVLMs, in the leaves of *C. violaceum* collected from different regions in China were evaluated, and the results are summarized in Figure 3. As shown in Figure 3, the DPPH radical scavenging activities of CVLMs ranged from 28.02 ± 0.93 to 92.55 ± 1.81 µmol Trolox/g, the ABTS radical scavenging activities of CVLMs ranged from 21.81 ± 1.72 to 81.12 ± 1.39 µmol Trolox/g, and the FRAPs of CVLMs ranged from 32.56 ± 1.30 to 95.77 ± 2.01 µmol Trolox/g, respectively. In each test, the significantly (*p* < 0.05) highest in vitro antioxidant activity was found in CVLM-C, followed by lower activity in CVLM-B and CVLM-A, and the lowest activity in CVLM-D, which might be attributed to the levels of phenolic compounds in these plants [35,36,41]. The results showed that CVLMs exert strong in vitro antioxidant activities, which are higher than those of various herbs and plants, such as *Astragalus membranaceus* (ABTS: 9.12 ± 0.11 µmol Trolox/g), *Agadtacge rygisa* (ABTS: 15.07 ± 0.51 µmol Trolox/g), *Amomum villosum* Lour (ABTS: 67.67 ± 2.33 µmol Trolox/g), *Camellia japonica* L. (DPPH: 30.05 ± 2.37 µmol Trolox/g; ABTS: 17.62 ± 1.12 µmol Trolox/g; FRAP: 87.96 ± 1.28 µmol Trolox/g), *Bombaxceiba* (DPPH: 67.21 ± 3.82 µmol Trolox/g; ABTS: 39.09 ± 0.88 µmol Trolox/g; FRAP: 86.74 ± 0.94 µmol Trolox/g), *Dianthus caryophyllus* (DPPH: 51.13 ± 3.82 µmol Trolox/g; ABTS: 10.03 ± 1.05 µmol Trolox/g; FRAP: 48.55 ± 1.38 µmol Trolox/g), and *Eriobotrya japonica* (DPPH: 55.52 ± 4.40 µmol Trolox/g; ABTS: 50.14 ± 1.13 µmol Trolox/g; FRAP: 99.21 ± 1.03 µmol Trolox/g) [20,21]. Therefore, the high levels of antioxidant activities of CVLMs from the leaves of *C. violaceum* implies that they could be used as natural antioxidants in industrial applications.

#### 3.3.2. Correlations between In Vitro Antioxidant Activities and Phenolic Compounds of CVLMs

Pearson’s correlation coefficient analysis was carried out to investigate the relationships between the in vitro antioxidant activities and the methanolic extractions from the leaves of *C. violaceum*. As shown in Table 4, highly positive correlations were detected between the in vitro antioxidant activities (including DPPH radical scavenging activities, ABTS radical scavenging activities and FRAP) and TPC in the leaves of *C. violaceum*, and highly positive correlations were also detected between the in vitro antioxidant activities and TFC in the leaves of *C. violaceum*. Results were in accordance with some reports that TPC and TFC in plants are closely correlated with their in vitro antioxidant activities [22,42,43], suggesting that the phenolic compounds in the leaves of *C. violaceum* are the major contributors to their in vitro antioxidant activities. Furthermore, two major individual phenolic compounds (chlorogenic acid and rutin) in CVLMs were also positively correlated with their in vitro antioxidant activities. Meanwhile, results were in accordance with previous studies that chlorogenic acid and rutin were closely correlated with the in vitro antioxidant activities [42,44].

### 3.4. In Vitro Antioxidant Activities of CVLPs

#### 3.4.1. In Vitro Antioxidant Activities

**Figure 4:**
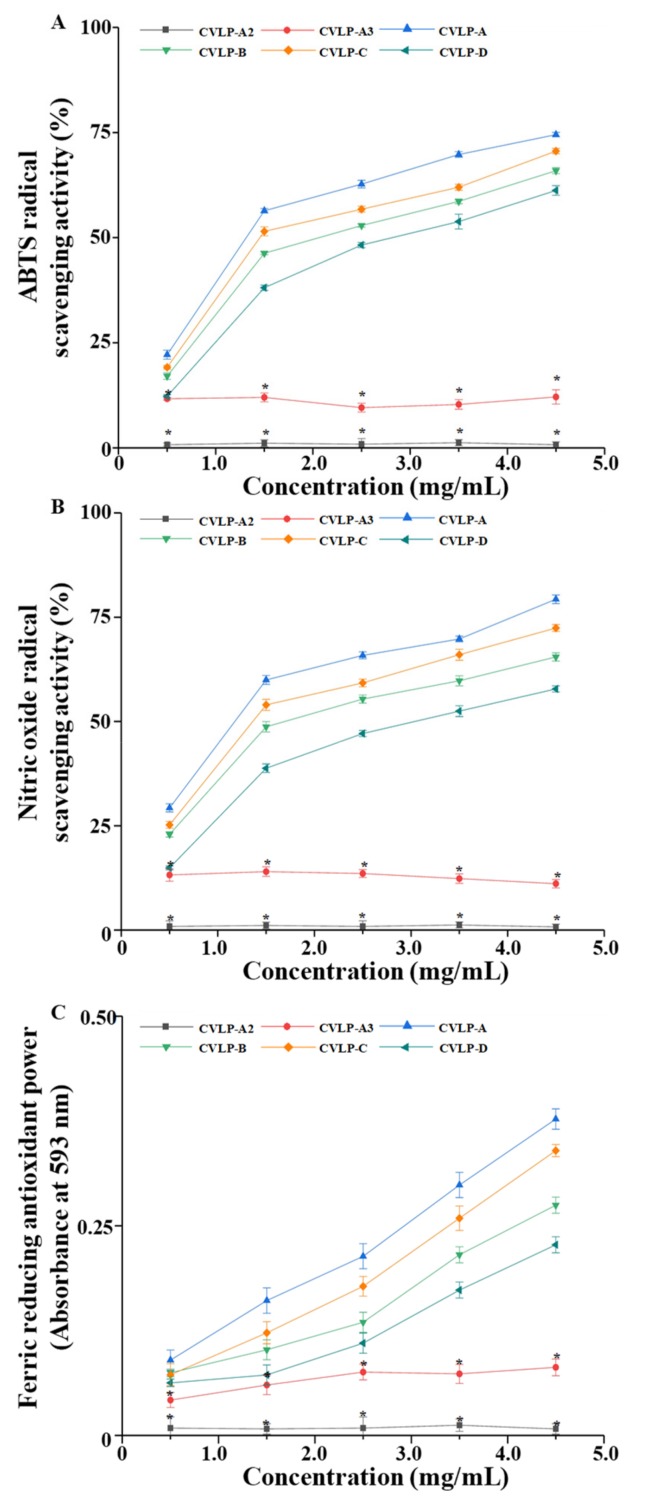
ABTS radical scavenging activities (**A**), nitric oxide (NO) radical scavenging activities (**B**) and ferric reducing antioxidant powers (**C**) of CVLPs and their acidic and enzymatic hydrolysates **CVLP-A**, **CVLP-B**, **CVLP-C and CVLP-D**, crude polysaccharides extracted from the leaves of *C. violaceum* collected from Chengdu, Langzhong, Bazhong and Guangyuan, respectively. **CVLP-A2**, partial acidic hydrolysates of CVLP-A; **CVLP-A3**, pectinase enzymatic hydrolysates of CVLP-A. Values represent mean ± standard deviation. Significant (*p* < 0.05) differences of ABTS radical scavenging activities, NO radical scavenging activities and FRAP between CVLP-A, CVLP-A2 and CVLP-A3 at the same concentration are shown by *; Statistical significances were determined by A.N.O.V.A. and Duncan’s test.

Recently, natural polysaccharides have drawn much interest as natural antioxidants due to their high antioxidant activities and low toxicity and side effects [7,8]. Previous studies have shown that polysaccharides in the roots of *C. violaceum* exhibit strong antioxidant activities [8,45]. However, the antioxidant activities of the leaves of *C. violaceum* have seldom been explored. Meanwhile, many polysaccharides extracted from leaves showed remarkable antioxidant activities [46,47,48]. Furthermore, in order to study the in vitro antioxidant activities of CVLPs and their possible structure–bioactivity relationships, it is necessary to use methods that are different from the methods used for CVLMs. Therefore, the in vitro antioxidant activities, including DPPH radical scavenging activities, NO radical scavenging activities, and FRAPs of CVLPs in the leaves of *C. violaceum* collected from different regions, were evaluated and are summarized in Figure 4A–C. The results showed the ABTS radical scavenging activities, NO radical scavenging activities and FRAPs of CVLPs exhibited a dose-dependent response. In addition, at the concentration of 4.5 mg/mL, the ABTS radical scavenging activities of CVLP-A, CVLP-B, CVLP-C and CVLP-D ranged from 61.22% to 74.50%. At the concentration of 4.5 mg/mL, the NO radical scavenging activities of CVLP-A, CVLP-B, CVLP-C and CVLP-D ranged from 57.82% to 79.31%. Additionally, at the concentration of 4.5 mg/mL, the FRAPs of CVLP-A, CVLP-B, CVLP-C and CVLP-D ranged from 0.22 to 0.38. As shown in Figure 4A–C, the significantly (*p* < 0.05) strongest in vitro antioxidant activities were found in CVLP-A, followed by weaker activities in CVLP-C and CVLP-B, and the weakest activity in CVLP-D. Indeed, the IC_50_ of ABTS radical scavenging activities of CVLP-A, CVLP-B, CVLP-C and CVLP-D were determined to be 1.47 mg/mL, 2.02 mg/mL, 1.84 mg/mL, and 2.80 mg/mL, respectively. In addition, the IC_50_ of NO radical scavenging activities of CVLP-A, CVLP-B, CVLP-C and CVLP-D were determined to be 1.18 mg/mL, 1.96 mg/mL, 1.54 mg/mL, and 2.97 mg/mL, respectively. Results further confirmed that CVLP-A exerted the strongest in vitro antioxidant activities among all tested samples, which might be attributed to its higher molecular weight and uronic acid content [3,39,40]. Furthermore, due to the existence of electrophilic groups, such as keto or aldehyde groups, in acidic polysaccharides, the liberation of hydrogen from O–H bonds is accelerated, and their radical scavenging activities are therefore improved [41]. Moreover, the IC_50_ of ABTS and NO radical scavenging activities of BHT (positive control) were measured to be 0.33 mg/mL and 0.27 mg/mL, respectively. Meanwhile, at the concentration of 1.0 mg/mL, the FRAP of BHT was determined to be 1.01. The results indicated that the in vitro antioxidant activities of CVLPs were lower than that of BHT. However, compared with the pectic-like polysaccharides extracted from other plants, such as *Suaeda fruticosa* [46], *Ziziphus jujuba* [49], *Lycium barbarum* [50], and okra [7], CVLPs showed stronger in vitro antioxidant activities, such as ABTS and NO radical scavenging activities. The results showed that CVLPs exhibited potent antioxidant activities, and have a strong potential for development as natural antioxidants for industrial applications.

#### 3.4.2. Correlations between the Glycosidic Linkage of CVLPs and Their In Vitro Antioxidant Activities

It has been reported that bioactivities, such as the antioxidant activities of natural polysaccharides, were highly correlated with their chemical structures, such as their glycosidic linkages [51,52,53]. Therefore, in order to better understand the structure–bioactivity relationship of CVLPs, the effects of glycosidic linkages on their antioxidant activities were investigated. As shown in Figure 4A–C, compared with CVLP-A, the ABTS radical scavenging activities, NO radical scavenging activities, and FRAPs of CVLP-A2 (the partial acidic hydrolysates of CVLP-A) barely existed. The results suggested that the polysaccharides fractions of CVLPs were the main contributors towards their in vitro antioxidant activities. However, considering the randomness of acidic hydrolysis of polysaccharides, the enzymatic degradation of CVLPs was performed to investigate the effects of the specific glycosidic linkages of CVLPs on their in vitro antioxidant activities. Meanwhile, according to the constituent monosaccharides of CVLPs, pectinase was selected to specifically degrade the α-1,4-D-galactosiduronic linkages in CVLPs. As shown in Figure 4A–C, compared with CVLP-A, the ABTS radical scavenging activities, NO radical scavenging activities and FRAPs of CVLP-A3 (the pectinase enzymatic hydrolysates of CVLP-A) decreased significantly, suggesting that its in vitro antioxidant activities were highly correlated with the α-1,4-D-galactosiduronic linkage of CVLPs. The results suggested that pectic-polysaccharides are the major contributors toward the in vitro antioxidant activity of CVLPs. Furthermore, the remaining in vitro antioxidant activities might be attributed to the existence of other types of polysaccharides in CVLPs [10,46]. In order to further reveal the structure–bioactivity relationships of CVLPs, the purified CVLPs will be further prepared to evaluate their structural features, and in vitro and in vivo antioxidant activities.

## 4. Conclusions

In this study, the major phenolic compounds in the methanolic extracts from the leaves of *C. violaceum* (CVLMs) collected from different regions were identified as chlorogenic acid and rutin, which ranged from 1.22 ± 0.03 to 2.87 ± 0.04 mg/g DW and from 2.25 ± 0.04 to 4.03 ± 0.05 mg/g DW, respectively. Meanwhile, the extraction yields of crude polysaccharides from the leaves of *C. violaceum* ranged from 4.73% to 5.41%. The CVLPs largely consisted of galacturonic acid, glucose, galactose, and arabinose, suggesting the existence of pectic-like polysaccharides. The results are beneficial for understanding the chemical properties of CVLMs and CVLPs. Furthermore, both CVLMs and CVLPs exhibited strong in vitro antioxidant activities. Chlorogenic acid and rutin were the major contributors to the in vitro antioxidant activities of CVLMs, and the in vitro antioxidant activities of CVLPs were closely correlated to their α-1,4-D-galactosiduronic linkages. The results suggest that the leaves of *C. violaceum* (byproducts) have great potential to be developed as natural antioxidants for industrial applications.

## Figures and Tables

**Table 1:** TPC, TFC and major individual phenolic compounds in CVLMs from the leaves of *C. violaceum* collected from different regions.

	TPC (mg GAE/g DW)	TFC (mg RE/g DW)	Chlorogenic Acid (mg/g DW)	Rutin (mg/g DW)
**CVLM-A**	9.33 ± 0.63^c^	6.32 ± 0.36^b^	1.89 ± 0.03^c^	2.88 ± 0.01^c^
**CVLM-B**	10.43 ± 0.71^b^	6.05 ± 0.29^b^	2.07 ± 0.02^b^	3.03 ± 0.04^b^
**CVLM-C**	12.55 ± 1.01^a^	7.97 ± 0.31^a^	2.87 ± 0.04^a^	4.03 ± 0.05^a^
**CVLM-D**	8.02 ± 0.27^d^	5.36 ± 0.22^c^	1.22 ± 0.03^d^	2.25 ± 0.04^d^

**TPC**, total phenolic content; **TFC**, total flavonoid content; **CVLM-A**, **CVLM-B**, **CVLM-C** and **CVLM-D**, methanolic extractions from the leaves of *C. violaceum* collected from Chengdu, Langzhong, Bazhong and Guangyuan, respectively. Values represent mean ± standard deviation, and superscripts a–d differ significantly (*p* < *0.05*) among CVLMs collected from different regions. Statistical significances were determined by A.N.O.V.A.

**Table 2:** Extraction yields, chemical compositions, molecular weights (*M_w_*) and polydispersities (*M_w_*/*M_n_*) of CVLPs.

	CVLP-A	CVLP-B	CVLP-C	CVLP-D
**Extraction yields (%)**	5.32 ± 0.22 ^a^	4.73 ± 0.14 ^b^	5.41 ± 0.27 ^a^	4.99 ± 0.13 ^b^
**Total polysaccahrides (%)**	81.08 ± 0.22 ^a^	80.92 ± 0.18 ^a^	82.08 ± 0.32 ^a^	81.83 ± 0.32 ^a^
**Total uronic acids (%)**	30.96 ± 0.22 ^a^	26.62 ± 0.17 ^c^	28.33 ± 0.31 ^b^	22.03 ± 0.49 ^d^
**Total Proteins (%)**	2.73 ± 0.11 ^a^	1.99 ± 0.16 ^a^	2.55 ± 0.25 ^a^	2.01 ± 0.15 ^a^
***M_w_*** **(×10^4^ Da)**				
**Fraction 1**	9.45 ± (3.25%) ^a^	7.12 ± (3.36%) ^c^	8.88 ± (3.17%) ^b^	5.37 ± (3.25%) ^d^
**Fraction 2**	1.21 ± (3.19%) ^a^	1.01 ± (4.11%) ^a^	1.15 ± (3.15%) ^a^	1.16 ± (3.13%) ^a^
***M_w_*** **/*M_n_* (polydispersity)**				
**Fraction 1**	1.43	2.00	1.79	1.48
**Fraction 2**	1.21	1.04	1.31	1.27

**CVLP-A**, **CVLP-B**, **CVLP-C and CVLP-D**, crude polysaccharides extracted from the leaves of *C. violaceum* collected from Chengdu, Langzhong, Bazhong and Guangyuan, respectively. Values represent mean ± standard deviation, and superscripts a–d differ significantly (*p* < 0.05) among CVLPs collected from different regions. Statistical significances were determined by A.N.O.V.A. and Duncan’s test.

**Table 3:** Molar ratios of constituent monosaccharides of CVLPs.

	Monosaccharides and Molar Ratios					
	Man	Rha	GalA	Glc	Gal	Ara
**CVLP-A**	1.00	3.03	6.54	4.55	6.39	3.11
**CVLP-B**	1.00	3.68	12.43	8.20	15.70	10.39
**CVLP-C**	1.00	5.29	14.19	6.45	17.68	10.48
**CVLP-D**	1.00	5.03	9.47	7.19	15.67	9.21

**CVLP-A**, **CVLP-B**, **CVLP-C and CVLP-D**, crude polysaccharides extracted from the leaves of *C. violaceum* collected from Chengdu, Langzhong, Bazhong and Guangyuan, respectively; **Man**, mannose; **Rha**, rhamnose; **GalA**, galacturonic acid; **Glc**, glucose; **Gal**, galactose; **Ara**, arabinose.

**Table 4:** Pearson’s correlation coefficient among TPC, TFC, chlorogenic acid, rutin and in vitro antioxidant activities of the leaves of *C. violaceum.*

	TPC	TFC	Chlorogenic Acid	Rutin	DPPH	ABTS	FRAP
**TPC**	1.000						
**TFC**	0.995 **	1.000					
**Chlorogenic acid**	0.953 **	0.984 **	1.000				
**Rutin**	0.954 **	0.968 **	0.994 **	1.000			
**DPPH**	0.893 **	0.906 **	0.921 **	0.959 **	1.000		
**ABTS**	0.903 **	0.902 **	0.905 **	0.936 **	0.935 **	1.000	
**FRAP**	0.892 **	0.900 **	0.914 **	0.976 **	0.944 **	0.959 **	1.000

**TPC, total phenolic content**; **TFC**, total flavonoid content; **DPPH**, DPPH radical scavenging activity; **ABTS**, ABTS radical scavenging activity; **FRAP**, ferric reducing antioxidant power; Correlation is significant at ** *p* < 0.01 level (tow-tailed), and the correlation relationships were analyzed by Pearson’s correlation coeffiecient analysis.
